# Sonographic and cytological predictors of thyroid malignancy using TIRADS and Bethesda systems: a cross-sectional study

**DOI:** 10.3389/fendo.2026.1804753

**Published:** 2026-04-23

**Authors:** Monier Towfeeq Ahmed Altaee, Maryam Issa Al-Ani, Rafea Jasim Hussien Al-Shammri, Mohammed Tareq Muter

**Affiliations:** Department of Surgery, College of Medicine, University of Baghdad, Baghdad, Iraq

**Keywords:** Bethesda system, fine needle aspiration cytology, multinodular goiter, papillary carcinoma, thyroid malignancy, thyroid nodules, TIRADS, ultrasound

## Abstract

**Background:**

Thyroid nodules are among the most common endocrine disorders and present a variable risk of malignancy. Accurate preoperative assessment using clinical, sonographic, and cytological parameters is essential for appropriate management.

**Methods:**

A prospective cross-sectional study was conducted on 70 patients presenting with thyroid complaints at Baghdad Teaching Hospital between January and June 2024. All participants underwent clinical evaluation, thyroid function testing, ultrasonography using the TIRADS classification, fine-needle aspiration cytology using the Bethesda system, and histopathological examination following surgery.

**Results:**

Multinodular goiter was the most prevalent thyroid condition in both sexes. Higher TIRADS grades, particularly Grade 5, and higher Bethesda categories were significantly associated with malignancy. Papillary carcinoma was the most frequent malignant histological type. Malignancy was more common among patients requiring re-aspiration. A positive family history and presentation with neck swelling were significantly associated with malignant nodules.

**Conclusion:**

The combined use of TIRADS and the Bethesda system provides reliable prediction of thyroid malignancy. Family history and neck swelling are important clinical indicators that should prompt closer surveillance and early diagnostic intervention.

## Introduction

Thyroid enlargement is a notable problem, and thyroid diseases are the most common endocrine condition. Structural abnormalities such as thyroid nodules, including diffuse multinodular goiter, as well as tumors (such as adenoma, papillary carcinoma, follicular carcinoma, and medullary carcinoma), represent common causes of benign and malignant thyroid enlargement (1–4).

Palpitations, anxiety, tremors, high blood pressure, and heat intolerance are some of the symptoms of hyperthyroidism. Conversely, hypothyroidism manifests as tiredness, constipation, cold intolerance, and weight gain. Hashimoto’s disease, follicular carcinoma, and toxic thyroid adenoma are more common in middle-aged women, whereas papillary thyroid carcinoma occurs predominantly in adults and shows a strong female predominance (5).

Thyroid diseases affect over 200 million people worldwide. 0.2–1.3% of people in iodine-sufficient areas have hyperthyroidism, 1–2% have hypothyroidism, and 47% have palpable thyroid nodules (6). Thyroid disorders were found in 24.3% of the 1,800 subjects in an Iraqi research study. Of the people studied, 18.1% had subclinical thyroid impairment, while 6.2% had overt thyroid dysfunction. Of the 170 patients on thyroid medication, 25.9% had overt thyroid illness and 31.8% had subclinical thyroid disease (7).

A thorough history, physical examination, thyroid function tests, and thyroid ultrasonography are essential for the evaluation of thyroid nodules. Fine-needle aspiration biopsy and cytological examination may be required depending on the clinical and ultrasonographic characteristics of the nodules. Thyroid function tests (TFT) measures serum T3, T4 and TSH. Calcitonin levels are reserved for evaluation of medullary thyroid cancer (8).

TIRADS is the gold standard for reporting on ultrasonography (USG), which is a safe and economical method of differentiating between benign and malignant thyroid nodules. However, the Bethesda system for cytopathology reporting supports Fine Needle Aspiration Cytology (FNAC), which is extremely sensitive and crucial for assessing neoplastic and non-neoplastic lesions (9).

### Aim of study

The purpose of this study is to investigate the frequency distribution of different conditions that manifest as thyroid enlargement and to closely examine their radiological and clinicopathological characteristics.

## Materials and methods

This study is prospective cross-sectional which had been carried out between January 1st and June 1st, 2024, at the Baghdad Teaching Hospital/Medical City. Patients with thyroid complaints who visited an outpatient clinic were included in the study. To concentrate the study on newly presenting, untreated cases, participants with known thyroid diseases, those who had previously undergone thyroid intervention due to benign or malignant conditions, or those who had already been diagnosed with thyroid malignancy were omitted.

Prior to a clinical examination that included an ENT exam, evaluation for retrosternal extension and lymphadenopathy, patients were first assessed based on their medical history, which included symptoms of hypo- or hyperthyroidism, history of radiation, family history, and prior history of thyroid problems. Thyroid function tests, complete blood count, ultrasonography, and fine needle aspiration were among the laboratory tests performed.

According to the TIRADS system, ultrasound features are given points between 0 and 3 in each category. The ultimate nodule categorization, which ranges from 1 to 5, where 1 is benign, 2 is not suspicious, 3 is mildly suspicious, 4 is moderately suspicious, and 5 is highly suspicious, is determined by adding these scores. Macrocalcifications, mixed cystic and solid composition, and hyper- or isoechoic echogenicity are characteristics that receive one point. Hypo-echogenicity, uneven borders, solid composition, and peripheral calcifications are characteristics that receive two points. Significant hypo-echogenicity, extra-thyroidal extension, a taller-than-wide shape, and s punctate echogenic foci are characteristics that earn three points (10).

The Bethesda score was used to evaluate thyroid nodules during fine needle aspiration. It is advised to repeat fine needle aspiration cytology (FNAC) and ultrasound for non-diagnostic results (grade 1), which have a 1% to 4% malignancy risk. Clinical follow-up is usually sufficient for benign findings (grade 2), where the risk is 0% to 3%. A repeat FNAC is recommended if the diagnosis is unusual or uncertain (grade 3), with a malignancy risk of 5% to 15%. A lobectomy is typically the result of nodules categorized as follicular neoplasms or suggestive for such (grade 4), which carry a 25% to 30% risk. If available, molecular genetic testing has been recommended for Bethesda 3 and 4 nodules before surgical intervention.

A total thyroidectomy or lobectomy, frequently with a frozen section for prompt diagnosis, is the standard procedure for nodules that are suspected for malignancy (grade 5), with a 60% to 75% chance. Lastly, nodules classified as malignant (grade 6), with a 97% to 99% chance, usually necessitate a lobectomy or complete thyroidectomy (11).

Medication was initially used to treat patients with abnormal thyroid profiles. After the medical treatment was finished, surgery was scheduled. After surgery, specimens were sent to a histopathology laboratory and preserved in formalin for histological investigation.

This study was approved by the Ethics Committee of the College of Medicine, University of Baghdad (Approval No. IBR-280; October 2023). All procedures were conducted in accordance with the Declaration of Helsinki. Written informed consent was obtained from all participants prior to enrollment. SPSS V.25 was used to analyze the data, and statistical tests were conducted with a 95% confidence level (p-value less than 0.05 is statistically significant).

## Results

**Figure 1:**
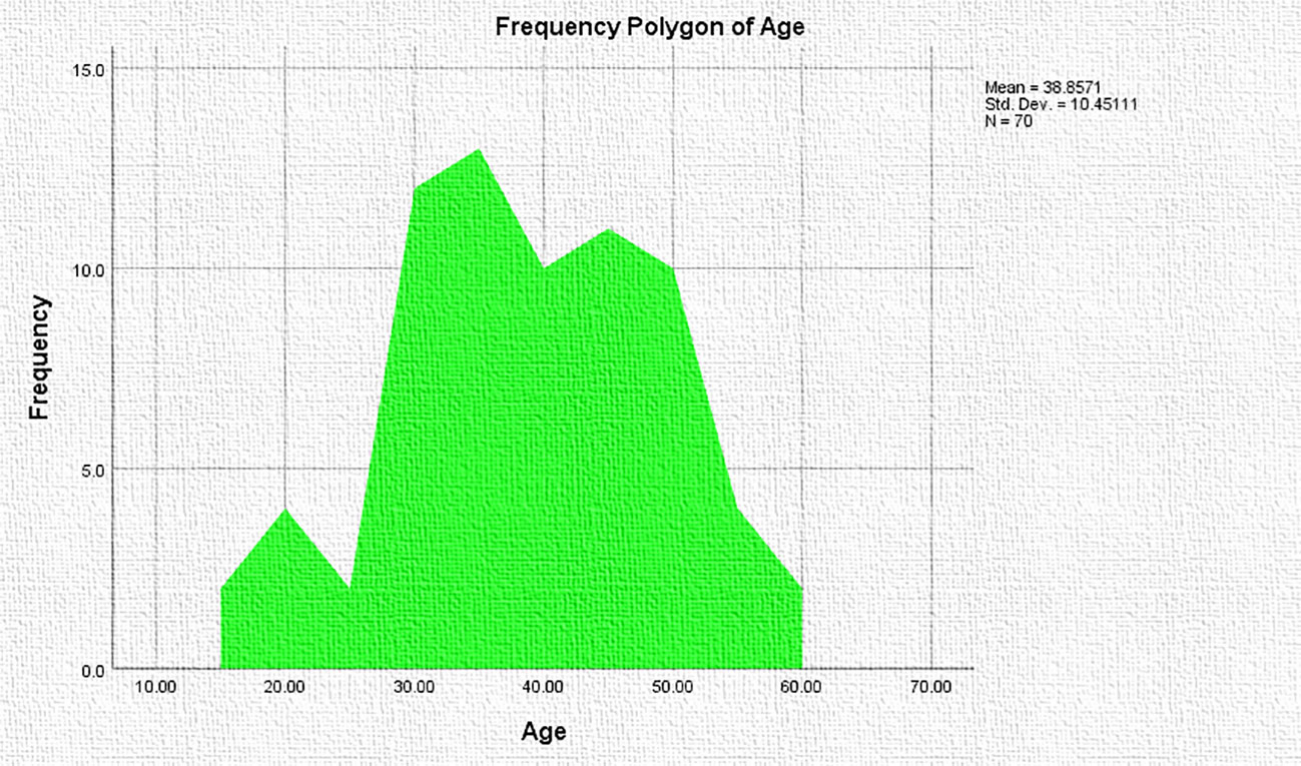
Frequency polygon showing the age distribution of study participants.

The age distribution is shown as a frequency polygon in **Figure 1**. There were 70 participants in total (63 females and 7 males), with a mean age of 37.79 years for females and 48.43 years for males. 75.7% of patients reported pressure discomfort as the most common clinical presentation, whereas 18.6% experienced neck swelling. Although they were rare, other symptoms were reported in 5.7% of individuals. Only three women were reported to have been exposed to radiation. 28.6% of men and 23.8% of women had positive familial history. As illustrated in **Table 1**, the majority of patients had never received treatment before, whereas lesser percentages had either thyroxine or carbimazole.

**Table 1:** Clinical characteristics of the participants.

Variable	Category	Female (n, %)	Male (n, %)	Total (n, %)
Age (years)	Mean	37.79	48.40	—
Presentation	Pressure symptoms	47 (74.6)	6 (85.7)	53 (75.7)
	Neck swelling	13 (20.6)	0 (0.0)	13 (18.6)
	Others	3 (4.8)	1 (14.3)	4 (5.7)
Radiation exposure	Yes	3 (4.8)	0 (0.0)	3 (4.28)
	No	60 (95.2)	7 (100.0)	67 (95.7)
Family history	Positive	15 (23.8)	2 (28.6)	17 (24.3)
	Negative	48 (76.2)	5 (71.4)	53 (75.7)
History of treatment	No	49 (77.8)	6 (85.7)	55 (78.6)
	Thyroxine	8 (12.7)	0 (0.0)	8 (11.4)
	carbimazole	6 (9.5)	1 (14.3)	7 (10.0)

Multinodular goiter (MNG) was more common in both sexes, with 85.7% of females and all males afflicted, according to sonographic and cytological features (**Table 1**). Males were more likely to score advanced grades (4 and 5) according to TIRADS grading. Details are shown in (**Table 2**). Bethesda cytology scores also varied, with Grade 3 being the most prevalent.

**Table 2:** Sonographic and cytological features by sex.

Variable	Category	Female (n, %)	Male (n, %)	Total (n, %)
Nodularity	MNG	54 (85.7)	7 (100.0)	61 (87.2)
	SNG	9 (14.3)	0 (0.0)	9 (12.8)
Left TIRADS	2	4 (6.7)	1 (14.3)	5 (6.4)
	3	20 (33.3)	2 (28.6)	22 (28.2)
	4	26 (43.3)	0 (0.0)	26 (33.3)
	5	10 (16.7)	4 (57.1)	25 (32.1)
Right TIRADS	2	5 (8.8)	0 (0.0)	5 (7.8)
	3	18 (31.6)	2 (28.6)	20 (31.2)
	4	27 (47.4)	4 (57.1)	31 (48.5)
	5	7 (12.3)	1 (14.3)	8 (12.5)
Isthmus TIRADS	4	1 (50.0)	0 (0.0)	1 (50.0)
	5	1 (50.0)	0 (0.0)	1 (50.0)
Bethesda category	2	21 (33.3)	2 (28.6)	23 (32.9)
	3	35 (55.6)	4 (57.1)	39 (55.7)
	4	1 (1.6)	0 (0.0)	1 (1.4)
	5	5 (7.9)	0 (0.0)	5 (7.1)
	6	1 (1.6)	1 (14.3)	2 (2.8)

**Figure 2:**
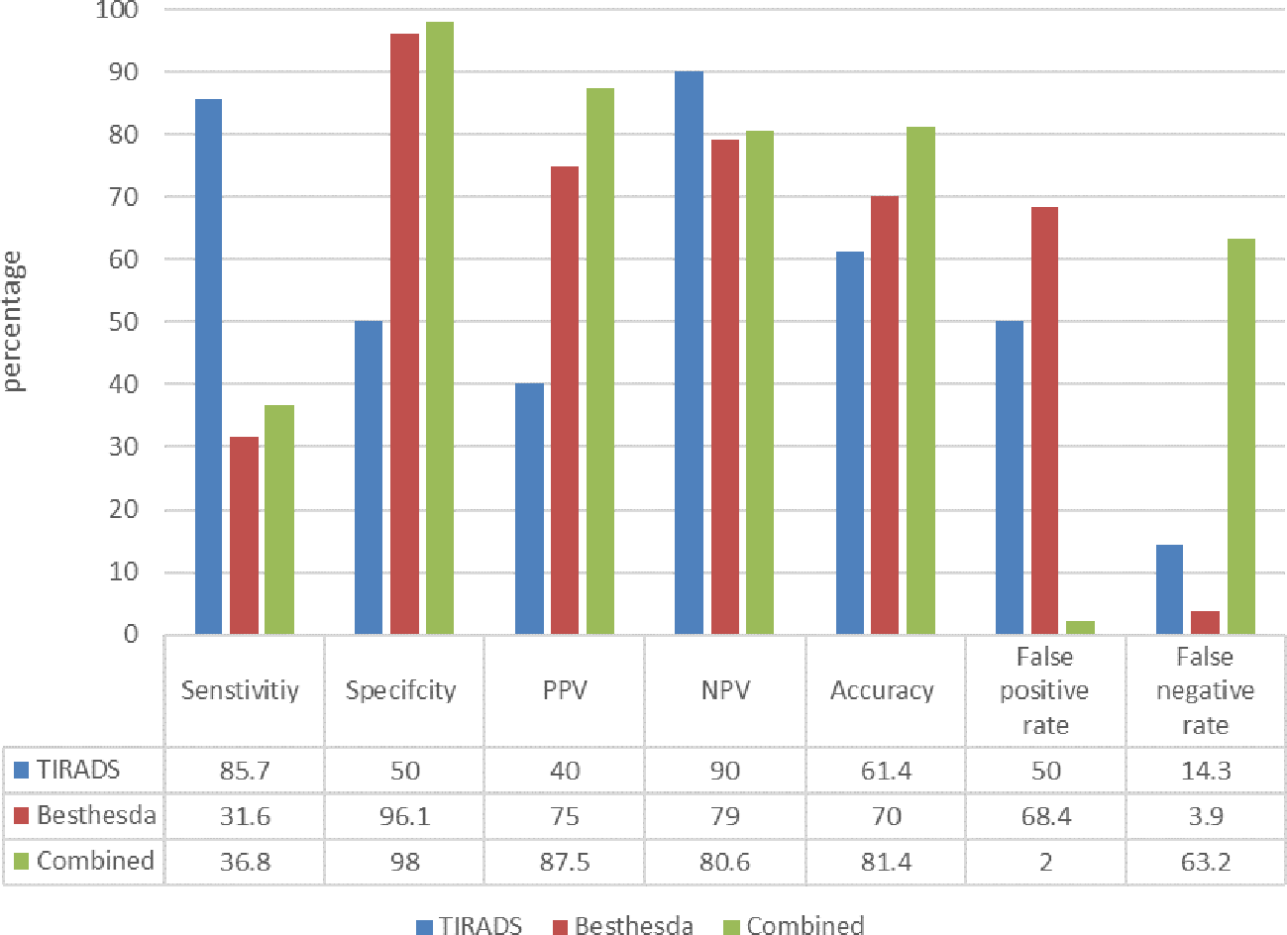
Comparison of diagnostic performance metrics for TI-RADS, Bethesda, and combined assessment.

Higher TIRADS grades were significantly linked to malignancy when stratified by histology, particularly TIRADS Grade 5, which was responsible for 69.2% of papillary carcinoma cases in the left lobe and 62.5% in the right lobe (**Table 3**). This pattern was confirmed by Bethesda cytology results, which showed that malignancy was more common in Grades 5 and 6. Malignant nodules were more common in patients who needed re-aspiration; in 62.5% of these cases, malignancy was found. In 26.2% of malignancies and 72.3% of benign nodules, total thyroidectomy was the preferred surgical procedure. The ultrasound-based TIRADS classification and cytological evaluation using FNAC demonstrated distinct diagnostic features in identifying papillary thyroid cancer, according to the diagnostic performance analysis. The majority of malignant nodules were accurately detected by ultrasonography, as evidenced by the high sensitivity of 85.7% when TIRADS category ≥4 was regarded as a positive result. Nonetheless, the specificity was rather low (50.0%), indicating that a significant percentage of benign nodules were also categorized as suspicious. Nodules classified as low-risk (TIRADS 2–3) were very likely to be benign, as indicated by the positive predictive value (PPV) of 40.0% and the negative predictive value (NPV) of 90.0%. In contrast, the Bethesda system demonstrates high specificity (96.1%) and low sensitivity (31.6%). The combined use of TI-RADS and Bethesda raised diagnostic capability, achieving the highest specificity (98%), positive predictive value (87.5%), and overall accuracy (81.4%) as in **Figure 2**.

**Table 3:** Radiological and cytological features according to histopathology.

Variable	Category	Papillary CA ^1^ (n, %)	Benign (n, %)
Left TIRADS	2	0 (0.0)	5 (100.0)
	3	3 (14.3)	18 (85.7)
	4	6 (24.0)	18 (72.0)
	5	9 (69.2)	4 (30.8)
Right TIRADS	2	1 (20.0)	4 (80.0)
	3	1 (5.3)	18 (94.7)
	4	9 (30.0)	20 (66.7)
	5	5 (62.5)	3 (37.5)
Isthmus TIRADS	4	1 (100.0)	0 (0.0)
Bethesda category	2	0 (0.0)	23 (100)
	3	13(33.3)	26 (66.7)
	4	1 (100.0)	0 (0.0)
	5	3 (60)	2 (40)
	6	2 (100.0)	0 (0.0)
Re-aspiration	Yes	5 (62.5)	3 (37.5)
	No	13 (22.0)	45 (76.3)
Surgery	Total thyroidectomy	17 (26.2)	47 (72.3)
	Lobectomy	0 (0.0)	0 (0.0)
	Subtotal thyroidectomy	1 (100.0)	0 (0.0) ^2^

Clinical characteristics that differentiated benign from malignant nodules are highlighted in (**Table 4**). 50% of those with cancer reported having a family history, and a positive family history was strongly linked to cancer (P = 0.02). Compared to pressure perceptions, which had a weaker correlation, presentation with neck swelling was more likely to be associated with malignancy (54.5%, P = 0.05). Other factors, on the other hand, did not exhibit statistically significant correlations with malignancy, including sex, thyroid status, history of radiation exposure, and nodularity type.

**Table 4:** Clinical features distinguishing benign and malignant thyroid nodules.

Variable	Category	Papillary CA^3^ (n, %)	Benign (n, %)	P-value
Sex	Female	16 (27.1)	43 (72.9)	0.935
	Male	2 (28.6)	5 (71.4)	
Presentation	Pressure symptoms	12 (23.5)	39 (76.5)	0.05
	Neck swelling	6 (54.5)	5 (45.5)	
	Others	0 (0.0)	4 (100.0)	
Radiation	Yes	1 (33.3)	2 (66.7)	0.809
	No	17 (27.0)	46 (73.0)	
Family history	Positive	8 (50.0)	8 (50.0)	0.019
	Negative	10 (20.0)	40 (80.0)	
Thyroid status	Euthyroid	15 (29.4)	36 (70.6)	0.206
	Hyperthyroid	0 (0.0)	7 (100.0)	
	Hypothyroid	3 (37.5)	5 (62.5)	
Treatment	No	15 (29.4)	36 (70.6)	0.206
	Thyroxine	3 (37.5)	5 (62.5)	
	carbimazole	0 (0.0)	7 (100.0)	
Nodularity	MNG ^4^	16 (27.6)	42 (72.4)	0.878
	SNG ^5^	2 (25.0)	6 (75.0)	

## Discussion

The clinicopathological and imaging characteristics of thyroid swellings are explored in this study, with a focus on the use of FNAC and ultrasound in the diagnosis and treatment of thyroid nodules. MNG is prevalent in both male and female patients, consistent with global trends. In this cohort, MNG was diagnosed in 85.7% of females and 100% of males, proving that it is common in both sexes (12).

The imaging results of the current study also provide important information. Calcifications and vascularity were the two primary factors used to differentiate between benign and concerning nodules; suspicious calcifications were more commonly associated with malignancy. These findings align with global studies that demonstrate the value of ultrasonography in guiding FNAC and subsequent therapy (13, 14).

The diagnostic utility of FNAC, which is still the cornerstone of thyroid nodule evaluation, was validated by this research. The majority of patients in this study had benign lesions (Bethesda grade 2). Hariprasad et al. reported that 73.58% of the cases were benign and 26.42% were malignant (15).

Similarly, Basharat et al. (16) evaluated 50 patients with thyroid swellings and discovered that 46% of them were benign, 44% had intermediate FNAC results, and 10% were cancerous. In research conducted by Padmawar et al. (17), 57 patients underwent FNAC; 89.47% of them were benign, while 10.52% were malignant.

According to our results that have been achieved in regards to diagnostic accuracy of TIRADS and Bethesda system the main limitation of TIRADS is low specificity and high false-positive rate which results in tendency to classify many benign nodules as suspicious or malignant, leading to a high rate of unnecessary FNAC procedures or even further investigations. Although TIRADS being highly sensitive (85.7%) and with low false-negative rate (14.3%), indicating it is ideal for identifying malignancy, its low specificity limits its diagnostic accuracy.

On the other hand, Bethesda system has very low sensitivity and high false-negative rate. This suggests that the Bethesda system may miss a considerable number of malignant cases, raises concerns about the reliability of this system as an independent screening tool for detecting malignancy. However, it has very high specificity (96.1%), suggesting that positive results are highly reliable.

Interestingly, family history has been shown as a remarkable predictor of cancer. Patients with a positive family history of thyroid disease had a higher incidence of cancer, which is consistent with earlier studies that have highlighted the significance of genetic vulnerability in the development of thyroid cancer (15). This conclusion suggests that patients with a family history of thyroid disease should be closely monitored and that early diagnostic therapy should be considered.

Similar to findings from the UAE (18), Saudi Arabia (19), and Yemen (20), this study found that papillary thyroid cancer was the most common type, followed by follicular thyroid cancer. In contrast, a study from Sudan which stated that follicular thyroid carcinoma was more common there (21). Follicular thyroid cancer listed third in Pakistan, with papillary thyroid cancer still the most prevalent histological type, followed by medullary carcinoma (22).

In addition, the malignancy rates of solitary thyroid nodules (STN) and multinodular goiter (MNG) did not vary significantly, according to this study. Different malignancy rates have been documented in earlier studies on this topic. Malignancy rates were 15.2% in STN and 8% in MNG, according to Abu Eshy et al. (23), whereas Ajarma et al. mentioned higher rates of 41.1% for STN and 29.2% for MNG (24). Other researchers reported lower malignancy rates for STN at 4.7% and 6.1%, respectively, using fine-needle aspiration cytology (FNAC) as demonstrated by Belfiore et al. (25) and Deandrea et al. (26).

Dirikoc et al. recorded the highest malignancy rates of 46.3% for STN and 41.4% for MNG (27). Similarly, Frates et al. (28) and Rago et al. (29) pointed to the value of FNAC, showing malignancy figures of 14.8% and 3.9% for STN, respectively.

Although FNAC and ultrasonography are widely used in the evaluation of thyroid nodules, both diagnostic methods have inherent limitations. FNAC may produce false-negative results due to sampling errors or inadequate specimens, and it cannot reliably differentiate between follicular adenoma and follicular carcinoma. Additionally, ultrasound findings may overlap between benign and malignant nodules and are highly operator-dependent. Therefore, combining cytological and radiological assessment with clinical evaluation is essential to improve diagnostic accuracy.

In this study, no link was found between radiation exposure and risk of malignancy. The lack of association between radiation exposure and thyroid malignancy in this study may be related to the relatively small sample size, which could have limited the ability to detect a significant relationship. A systematic review explores the effects of low-dose ionizing radiation on thyroid function in occupationally exposed workers; it found a possible link between radiation exposure and thyroid dysfunction or disease (30). Several studies report a significant association between radiation exposure and thyroid cancer risk, including those by Lee et al. (31), Park et al. (32), and Prysyazhnyuk et al. (33), showing elevated incidence among medical and Chernobyl-exposed workers. In contrast, studies by Kitahara et al. (34) and Gudzenko et al. (35) found no statistically significant link between radiation dose and thyroid malignancy. A similar Iraqi study in Mosul by Abdulrazaq et al. found no significant relation with ionizing radiation (36).

In recent years, a rise in thyroid cancer cases in Iraq was reported similar to the worldwide observation which might be partly due to improved diagnostic practices, especially the frequent use of surgical histopathology. Many cases are detected at early stages, suggesting increased medical surveillance may be uncovering small, low-risk tumors. However, a true increase in incidence is also possible due to factors like environmental exposure, genetic predisposition, and changes in iodine intake (37, 38).

The main limitation of this study lies in the lack of advanced diagnostic tools such as molecular testing, which are increasingly being utilized for more precise risk stratification. Additionally, the study design did not incorporate long-term follow-up of patients, which could provide insights into the progression, recurrence, or outcomes of thyroid malignancies. Furthermore, the relatively small sample size and the single-center design of the study may limit the generalizability of the findings.

In conclusion, multinodular goiter (MNG) was the most prevalent thyroid condition, with pressure symptoms being the dominant clinical presentation. Papillary carcinoma was the most common malignancy, strongly associated with higher TIRADS and Bethesda grades, while a positive family history and neck swelling were significant clinical predictors of malignancy. The strong correlation between TIRADS scores and histopathological outcomes underscores the accuracy of this scoring system in predicting malignancy. Additionally, the findings support the need for closer monitoring of patients with a positive family history of thyroid disease, given the increased risk of malignancy in this group. Future studies with larger sample sizes and long-term follow-up are recommended to further validate these findings and refine the diagnostic approach to thyroid swellings.

## Data Availability

The original contributions presented in the study are included in the article/supplementary material. Further inquiries can be directed to the corresponding author.

## Footnotes

1 Carcinoma.

2 Percentages are calculated within each histopathological category.

3 Carcinoma.

4 *Multinodular goiter*.

5 *Solitary nodular goiter*.
